# Design, Development and Testing of a Monitoring System for the Study of Proton Exchange Fuel Cells and Stacks

**DOI:** 10.3390/s23115221

**Published:** 2023-05-31

**Authors:** Milena L. Zambrano H, Antonio José Calderón, Manuel Calderón, Juan Félix González, Reinhardt Pinzón, José Rogelio Fábrega Duque

**Affiliations:** 1 Victor Levi Sasso Campus, Universidad Tecnológica de Panamá, Centenario Highway, Ancon, Panama 0819-07289, Panama; milena.zambrano@utp.ac.pa (M.L.Z.H.);; 2 Department of Electrical Engineering, Electronics and Automation, University of Extremadura, Elvas Avenue, n/n, 06006 Badajoz, Spaincalgodoy@unex.es (M.C.); 3 Department of Applied Physics, University of Extremadura, Elvas Avenue, n/n, 06006 Badajoz, Spain; jfelixgg@unex.es; 4 Sistema Nacional de Investigación (SNI), SENACYT, Panama 01001, Panama; 5 Estudios Multidisciplinarios de Ciencias y Tecnologías de la Ingeniería (CEMCIT-AIP), El Dorado, Panama P.O. Box 0819-07289, Panama

**Keywords:** cell voltage, stack, data acquisition, fuel cells, LabView

## Abstract

This article is about the design, development and validation of a new monitoring architecture for individual cells and stacks to facilitate the study of proton exchange fuel cells. The system consists of four main elements: input signals, signal processing boards, analogue-to-digital converters (ADCs) and a master terminal unit (MTU). The latter integrates a high-level graphic user interface (GUI) software developed by National Instruments LABVIEW, while the ADCs are based on three digital acquisition units (DAQs). Graphs showing the temperature, currents and voltages in individual cells as well as stacks are integrated for ease of reference. The system validation was carried out both in static and dynamic modes of operation using a Ballard Nexa 1.2 kW fuel cell fed by a hydrogen cylinder, with a Prodigit 32612 electronic load at the output. The system was able to measure the voltage distributions of individual cells, and temperatures at different equidistant points of the stack both with and without an external load, validating its use as an indispensable tool for the study and characterization of these systems.

## 1. Introduction

Fossil-based fuel sources are well-documented causes of global warming, due to the emission of carbon dioxide and other greenhouse gases that are causing devastating consequences for the environment [1].

In the most recent report by global energy experts [2], they indicate that despite the fact that the percentages of use of renewable energies have been growing, the global electrical transition must maintain very high levels of growth rates to replace carbon and reduce emissions.

New global policies state objectives such as the adoption of measures to reduce dependence on such fuel sources [3]. The United Nations Climate Change Conference held in Paris (COP21) in 2015 proposed the reduction of greenhouse gas emissions with the goal of limiting global temperature change to 1.5 °C [4]. To meet these objectives, research into the development of alternative energy sources to reduce global dependence on fossil fuels is required.

Hydrogen (H2) has the potential to help resolve the issue of clean energy, as it is considered a clean energy source that can be generated easily from primary energy sources and stored for future use to fill gaps in the power grid when other sources of energy, such as solar energy, are not available [5]. Fuel cells are electrochemical systems that use hydrogen and oxygen to produce electric energy. They work based on an oxidation–reduction reaction in which hydrogen acts as a fuel and circulates in the anode (negative electrode), while an oxidising agent, generally oxygen or air, is continuously fed into the cathode (positive electrode). In these types of proton exchange membrane fuel cells (PEMFC), which take hydrogen and oxygen as inputs, water is the only by-product generated [6].

Apart from the electrodes, these systems also consist of an electrolyte, a gas diffusion layer, cooling channels and a proton exchange membrane. Many systems are often added to a fuel cell to improve its performance, for example, cooling systems, filtering systems, gas feeding systems and so on. 

To incorporate these systems into the power grid, it is important to develop techniques to help characterize the system and diagnose any faults, and which permit these systems to operate optimally and prolong the lifespan of the product. Notable operating factors that affect performance are the degradation of components, the non-linearity of fuel distribution across the elements, impurities, air composition, catalysts, the management of water circulation, temperature, membrane humidity, and voltage reversal in the cells. [7].

## 2. Previous Works

Dynamic system modelling and diagnostics can be carried out using experimental techniques and models. This is achieved by comparing the results of the behaviour measured by sensors with the results predicted by the models [8]. Experimental techniques contain a set of tests such as Nyquist plots [9], fuel pressure monitoring [10], polarization curves [11], current density measurements [12,13], and gas chromatography [14], to name a few.

An experimental study of a PEM fuel cell is carried out using polarization curves, which are compared with the results of a steady-state model developed in MATLAB. The model predicts an increase in the performance of PEMFC with increased operating temperature, pressure, and reagent moisture [15].

A method of applying the EIS for the diagnosis of fuel cell degradation through an algorithm that can be carried out within the typical control of a fuel cell is investigated. The advantage of this method is that it is not necessary to carry out a complete impedance spectroscopy, which allows its use in systems that are in normal operating mode [16].

A methodology based on short-time analysis of voltage and pressure signals is used to quantify the effects of flooding and drying within proton exchange membrane fuel cell (PEMFC) systems. For the detection of fluctuations in these parameters in cases of flooding and drying, a standard deviation is used. With this study, a reliable tool is obtained for the study of these systems [17].

An important parameter in the study of these systems is the stack voltage, which can be used to detect a drop in performance and aid in the preventative testing of these electrochemical systems [18]. On this basis, many researchers have worked on fault detection based on total cell voltage, such as [19], who designed a monitoring system based on LabView and a myRIO embedded system, with which they monitored and obtained the data of a fuel cell in a vehicle. With this tool, they managed to obtain the current and voltage of the stack as well as measure these parameters at the battery, motor, and DC converter. Benouioua et al. [20] proposed the development of a fuel cell characterization tool based on singularity spectrum voltage (VSS), a pattern that can be estimated by the evolution of the voltage of the cell, as indicated in [21]. In this study, the authors obtained and verified system state indicators under different modes of operation (linked with better or worse air diffusion, and with higher or lower membrane hydration).

Although the total cell voltage evolution technique can be an indicator for fault detection, Ashraf Khorasani et al. [9] indicated that individual cell voltage measurements lead to much higher precision [22,23,24,25,26,27], as these voltages change faster and are more sensitive to cell conditions, which leads to earlier problem detection before the total fuel cell voltage is significantly altered. Moreover, voltages can reduce in any cell as a result of internal events, such as a water drop that restricts the flow of gas, or a hole in a membrane [28].

Adopting the unit cell voltage measurement method, the Group for Research on the Use of Biomass and Renewable Energy (or GAIRBER, its acronym in Spanish) of the University of Extremadura in Spain has designed, developed, and validated a real-time monitoring system for the diagnosis and detection of fuel cell failures at a cell level, capable of being replicated for fuel cells up to 120 cells [7].

Presented a free software tool for real-time simulation and observation of the different variables associated with an 80-cell polymer electrolyte fuel cell. For this purpose, they used a semi-empirical mathematical model developed from the manufacturer’s data to predict its behaviour, which can be extrapolated to other types of fuel cells [22].

Vivas, De las Heras, Segura and Andújar [23] developed a “plug and play” prototype through a USB connection to the computer, which allows the monitoring of cell voltages and is applicable to an 80-cell stack with a maximum current of 10 A. This prototype, in addition to measuring cell voltages, also allows measurements of stack current and temperature with good accuracy.

Cai, Maike Ye, Quan and Quan [29] developed a monitoring system using an LTC6803 microcontroller that allows the measurement of up to 12 voltage signals in cells. This measurement setup is carried out using a cascade configuration until a total input of 135 cells, with which the experimental fuel cell operates, is reached.

Migliardini, Di Palma, Gaele and Corbo [24] conducted an experimental study with a fuel cell with 96 individual cells and a power of 6.2 kW, for which they performed a voltage uniformity analysis, both in a steady state and under dynamic conditions, detecting increasing uniformities at higher stack powers. Between cells, maximum variations well below 50 mV were observed. For dynamic conditions, at higher speeds and power jumps in time, a higher non-uniformity in the voltages between individual cells was observed, with differences of up to 100 mV between some of them.

Hu et al., 2018 [25] proposed a model to explain the co-current and voltage redistribution mechanism between cells, using the method of two-point measurements along a bipolar plate and assuming that there is a voltage variability along it due to non-homogeneous distributions of currents. In this study, it is defined that the distribution of failed cells can affect the current and voltage distribution of their neighbouring cells.

Boškoski, Debenjak, and Boshkoska [30] propose a method of evaluating a fuel cell by applying different water management faults, for which they develop a low-cost data acquisition device capable of performing single-cell measurement within an HD-8 fuel cell stack, produced by the Hydrogenics Corporation, with an 8.5 kW capacity and consisting of up to 90 cells.

Luo and Jian, 2019 [31] investigated using a PEM stack, a strategy to mitigate the starvation effect that occurs in cells due to a lack of hydrogen in operations, rapid load changes, and uneven flow distribution. To do this, they replaced the one-way purge valve with a three-way purge valve to allow extra hydrogen input to the stack. With this configuration, a 10.67% increase in stack power was obtained; therefore, it is concluded that there is an increase in cell voltage as the hydrogen content in the downstream anode flow channel increases. This technique reduces cell voltage fluctuations and improves cell uniformity. The measurements of individual cells are performed with multichannel measurement equipment (GM10, Yokogawa Electric Corporation, Tokyo, Japan) that allows up to 420 analogue inputs.

Huang, Zhao, and Jian [32] developed an acquisition system using a multichannel system (Smart DAC+GM, Yokogawa Electric Corp., Tokyo, Japan) capable of measuring 40 channels. With this system, they studied the behaviour of fuel cell voltage when starvation occurs, and proposed a technique to resolve this state, using hydrogen at high pressure from an anode outlet, which was effective in improving the performance of the dynamic operation of this system.

Giacoppo, Hovland, and Barbera [26] Performed operational performance tests with a fuel cell made up of two interconnected stacks to verify its performance in operations or variable load profiles over time, representative of different lunar surface exploration missions. They developed a monitoring system consisting of a test station manufactured by “Lynntech” for the control of different flow parameters (flow velocity, flow rate, temperature, and relative humidity). With this system, it was possible to obtain the voltage distribution along the fuel cell, making measurements for individual cells in open circuit operation and with a variable load, in addition to tracking the stack voltage and co-current. They observed a homogeneous distribution in cells, and on the other hand, demonstrated the high performance and potential of these systems in space applications.

Finally, we can mention techniques that use different diagnostic tools, together with measurements of voltages in individual cells, as is the case of Hua et al. [33], who proposed a methodology based on a polarization curve analysis and the EIS (electrochemical impedance spectroscopy) technique to obtain internal cell parameters, and as a last step, measurements in individual cells to support the first two techniques. With this methodology, information can be obtained regarding the voltage uniformity in cells and the performance obtained by means of the polarization curve and internal resistances of each of the cells that make up the stack.

Used polarization curves and measurements in the individual cells of a 20-cell open-cathode proton exchange fuel cell to investigate the performance of the stack, using a cell separator structure with multiple holes, which verifies an increase in the performance of the stack and exhibits an improvement in cell voltage uniformity in regions of higher currents [34].

This study is a new proposal or technique for monitoring fuel cells that in addition to measuring the voltage of the entire set (stack), allows the measurement of temperature, current, and voltage in cells. On the other hand, a routine has been integrated that makes it possible to isolate, through software, the cell with the best and greatest operating efficiency. The value of this study lies in the fact that it has a research orientation and can be used as a tool in studies on PEFC supervision, as well as to improve operational efficiency in these electrochemical systems.

The system comprises two subsystems: a hardware part, consisting of three DAQs (NI-USB 6225, 6008, and 6218) and different signal processing elements, and a second, software part, with a virtual instrument platform that allows for the storage and monitoring of data in real-time. This characterization and diagnosis tool aims to improve the efficiency of these systems, with the end goal of using fuel cells as a good renewable energy source. 

After this introduction, the next section discusses data acquisition systems and virtual instruments. The third section deals with the architecture, construction, and development of a system monitoring software. The fourth section shows a prototype applied to different tests, using a 1.2 kW fuel cell to verify its functionality. Finally, a few results and conclusions are discussed in the last section.

## 3. Description of Data Acquisition Systems (DAQ) and Virtual Instrumentation

**Figure 1:**
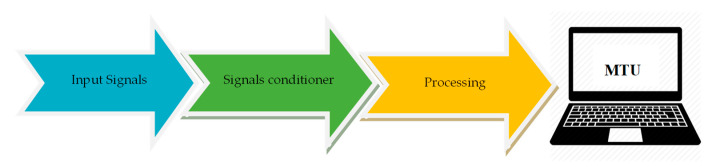
Description of data acquisition systems and virtual instrumentation.

The data acquisition system structure is based on different elements, as shown in Figure 1. The input signals have different parameters that are to be studied, and the signal conditioning part is used to correct and filter the input signal from the sensors or transducers. Signal processing elements allow the signals to be digitized and read from digital to analog converters (DACs) that transmit information to the unit named the MTU (master terminal unit), which then handles the manipulation, storage, and graphing of the data [35].

Many data acquisition systems work based on virtual instruments, created using a software application and programmed using a development platform. Unlike traditional instruments, these virtual instruments can be modified and updated as required to meet the needs of a particular experiment. Their development is achieved on a computer, making use of its vast computational resources to emulate a large variety of instruments such as multimeters, oscilloscopes, and signal analysers, with the option to add features such as numerical analysis, data visualization, storage, and processing, among others, using graphical representation on displays [36].

To implement the virtual instrumentation, a DAQ with analogue inputs and an analogue-to-digital converter (ADC) are required to measure and convert the analogue signals sent by the sensors to digital data that can be recognized by the MTU. This transmission requires a communication interface, which could be any one of RS-232, USB, or Ethernet, and a controller that facilitates communication between the DAQ and the MTU. In our study, the USB interface was used for communication between the DAQ and the MTU.

**Figure 2:**
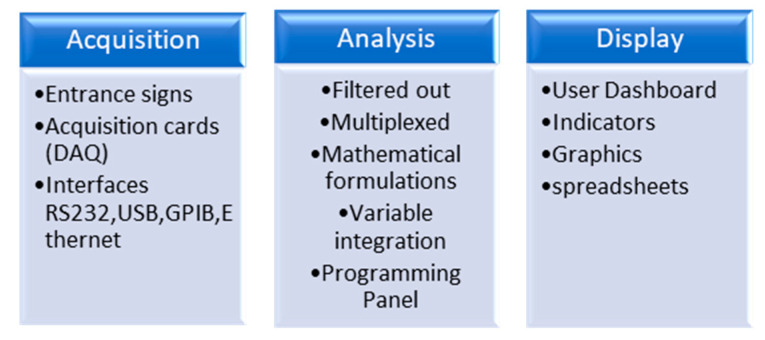
Elements of a virtual instrument.

Virtual instrumentation development also requires a programming environment that allows the creation of a software application, which is used for the creation of virtual instruments and within which various variable integrations, mathematical formulations, multiplexing, and demultiplexing are performed using different functions available in the application platform. It also includes the step of creating a graphic user interface that shows the graphs, data tables, and indicators that will help serve to monitor a specific process, as shown in Figure 2.

Different virtual instrument design software exists, of which the most recognized ones are MATLAB [37], Agilent-vee [38], My OpenLab [39], etc. Each one has its own characteristics for the creation and configuration of virtual instruments as needed to meet the requirements of a particular application. For this specific study, Laboratory Virtual Instrument Engineering Workbench (LabVIEW) was used as it is widespread, has support for numerous applications, and is considered the global reference in professional instrumentation [8]. It is also compatible with different hardware platforms, such as DAQs (for digital as well as analogue inputs and outputs), counters, digital multimeters, oscilloscopes, real-time embedded controllers, serial communication devices, motor controllers, cameras, FPGAs, microcontrollers, PLCs, etc. [40].

This platform was used to perform different studies in the area of fuel cells; especially worth mentioning is the voltage monitoring of individual cells [7], which was particularly developed with a system consisting of an Arduino that allowed the monitoring of voltages in a 7-cell PEM fuel cell.

On the other hand, [19] designed a GUI monitoring system based on LabView and a myRIO embedded system, with which they monitored and obtained data from a fuel cell in a vehicle showing stack voltage and current, as well as battery, motor, and DC–DC converter parameters. In [41], a module to monitor fuel cells and solar panels was developed in LabView. The paper [42] uses a data acquisition system implemented using LabView to conduct a feasibility study on the use of fuel cells in automobiles. The use of LabView as part of an experimental method to validate a hybrid prognosis and of a health management system for a PEM fuel cell is discussed in [43]. On the other hand, [44] developed a virtual graphical programming interface to monitor and measure the real-time impedance and operational stability of a fuel cell, using a National Instruments DAQ, non-invasively. All these research articles provide evidence that this tool has the capability to program and reprogram a virtual instrument [45].

## 4. Results

### 4.1. Development of the Monitoring System

A monitoring system was designed to acquire analogue signals as well as the voltages of each cell that comprise the stack voltage, cell temperatures, and stack temperatures. The DAQs have the potential to acquire a certain number of analogue signals, depending on the model. Based on this, it was necessary to use three different DAQs: the National Instruments NI USB-6225, NI USB-6218, and NI USB-6008. The NI USB 6225 and NI USB 6218 data acquisition devices measure at 32-bit and 16-bit resolutions, respectively. Inputs 1–40 of the NI USB-6225 acquired the 40 voltage signals from cells 1–40; the NI USB-6218 acquired, in the same order, the voltage signals from cells 41–47, and output 8 of the module was enabled to measure the battery current. The NI USB-6008 module was purchased to acquire the temperature signals.

This project requires 47 analogue inputs for each cell of the fuel cell. The NI USB-6225 possesses 40 common-mode analogue inputs. However, the common mode voltage of the NI E-Series DAQs, and specifically this model, is rated ±11 V maximum. Thus, although the individual cell voltages were a rather low 1 V per cell, the common mode voltage of the 40 cells of the fuel cell would be 40 V, which exceeds the rated voltage specifications of this DAQ. Although most of these DAQs possess an overvoltage protection of between 20 and 40 volts and would not actually damage the DAQ, this would lead to inaccurate and unpredictable readings [46].

**Figure 3:**
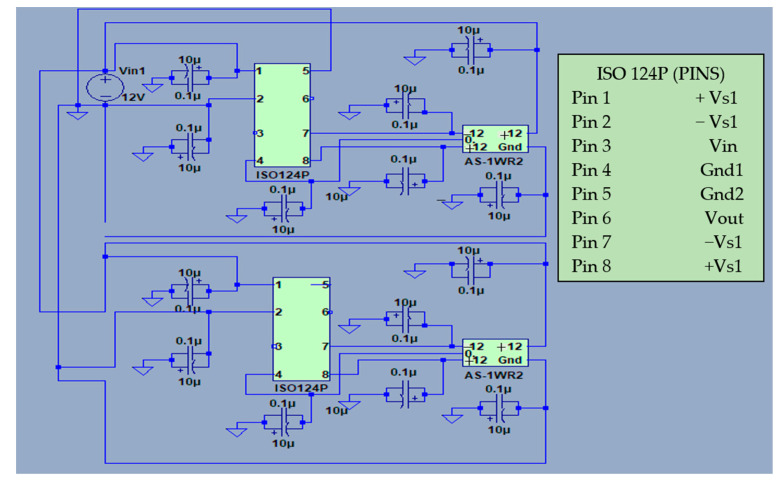
Schematic electrical circuit of two sections of the signal conditioning system.

To overcome this limitation, a module was specially designed (Figure 3) to isolate the ground terminals of each of the 40 analogue signals. This module makes use of ISO 124P precision isolation amplifiers to provide signal isolation. They have the distinct advantage of having unity gain, so the input and output signals have the same amplitude and need no modifications.

**Figure 4:**
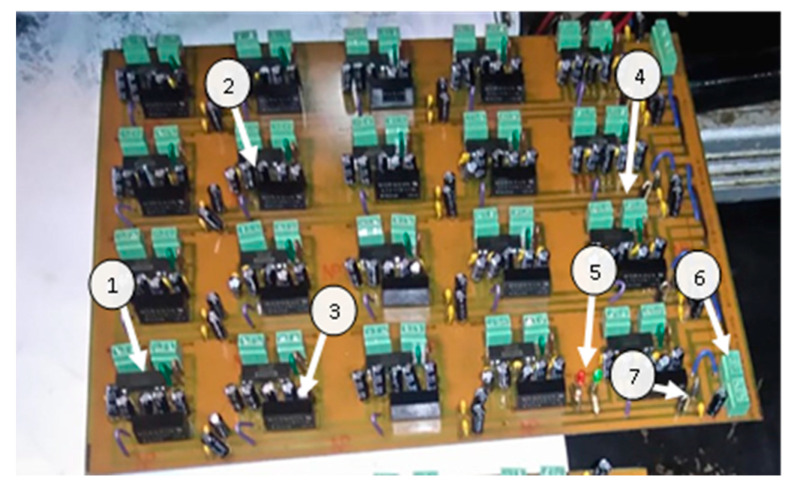
Signal conditioning board components. (1) isolation amplifier; (2) decoupling capacitor; (3) DC–DC converter; (4) I/O signal connectors; (5) LED polarity indicators; (6) +12 V and −12 V power input connectors; (7) zener diodes.

The components used for the design and construction of the ground isolation module consist of four main parts: the ISO 124P isolation amplifier, the DC–DC isolation converters, the decoupling capacitors, input/output connectors, and a Zener diode for reverse polarity protection, as shown in Figure 4. 

**Figure 5:**
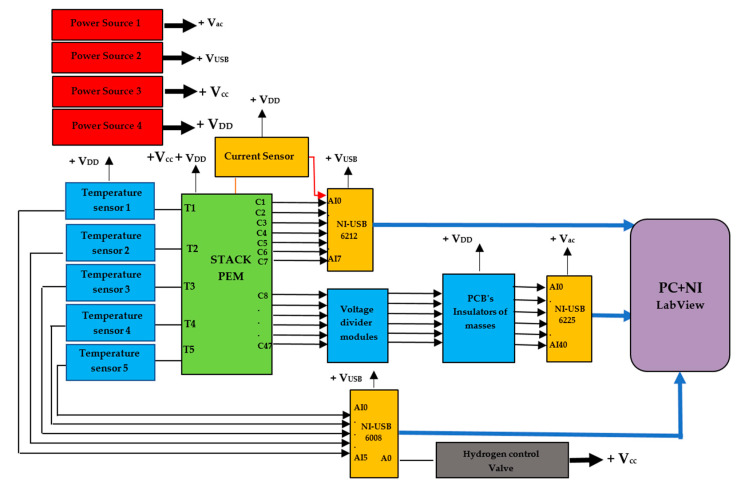
System block diagram with DAQs and other components.

The monitoring system consists of the three National Instruments DAQs (NI USB-6225, NI USB-6218, and NI USB-6008) mentioned previously. HTB-100 current sensors and LM-35 temperature sensors are used for current and temperature measurement, respectively. The LM35 temperature sensor used allows for the measurement of temperatures in a range from −55 °C to 150 °C. The sensor is calibrated to an accuracy of ±0.5 °C. Two ground isolation boards were made with the ISO124P isolation amplifiers, A_S-1WR2 isolated DC–DC converters, 1 µF ceramic, and 10 µF electrolytic capacitors, and 1N4005 rectification diodes, as seen in Figure 5.

### 4.2. Real-Time Monitoring System

**Figure 6:**
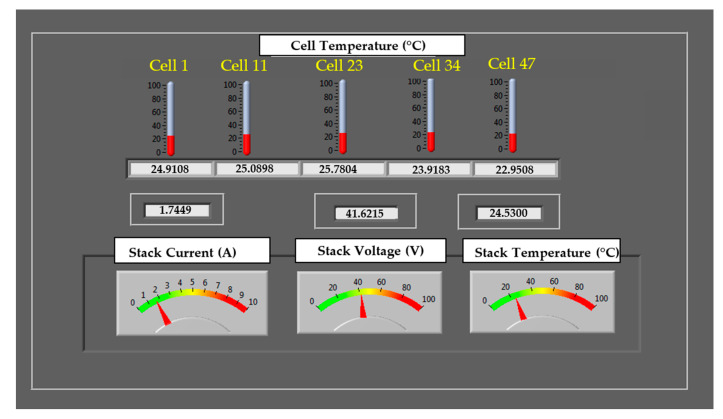
Designed GUI with numeric and graphics indicators.

A virtual interface was designed using different functions in LabView, with the objective of obtaining an acquisition routine into which the measurements from each of the three DAQs are incorporated. The GUI interface (Figure 6) contains numerical and graphical data with which the acquired data can be intuitively represented, allowing for visual access to all the system controls available in the system under study. This user interface includes a sampling time control, and bar graphs showing cell temperatures, and their corresponding numerical values. These measurements are made within a test time range of 100 milliseconds.

**Figure 7:**
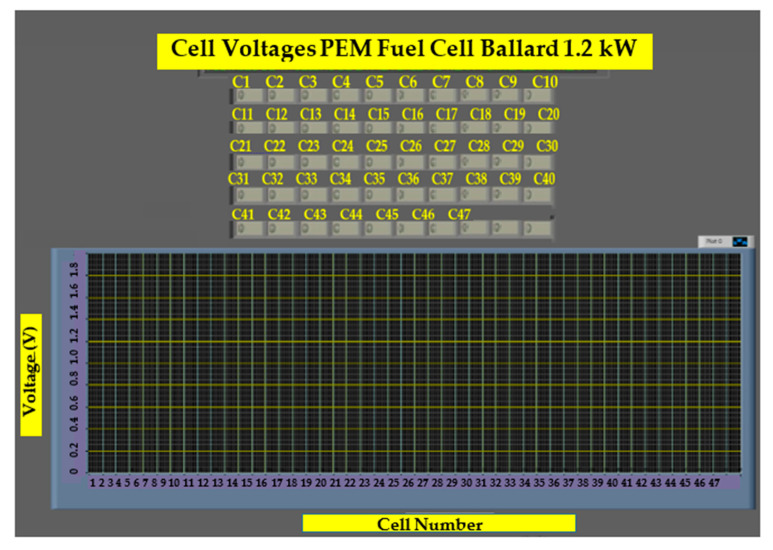
Voltage monitoring in the GUI.

In the second user panel shown in Figure 7, the GUI is shown with a table representing the voltage levels of each cell in a Ballard Nexa 1.2 kW, alongside bar graphs showing the voltage distribution across the entire stack.

**Figure 8:**
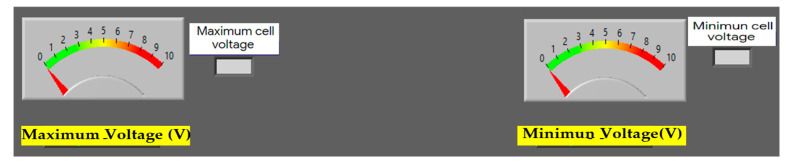
Real-time maximum and minimum cell voltage indicators.

An important aspect in the study of this system is the ability to detect cells with higher and lower efficiency, for which there are two indicators that show the cells with the highest and lowest voltage, as shown in Figure 8. This helps to quickly detect and identify any cells that may be malfunctioning.

**Figure 9:**
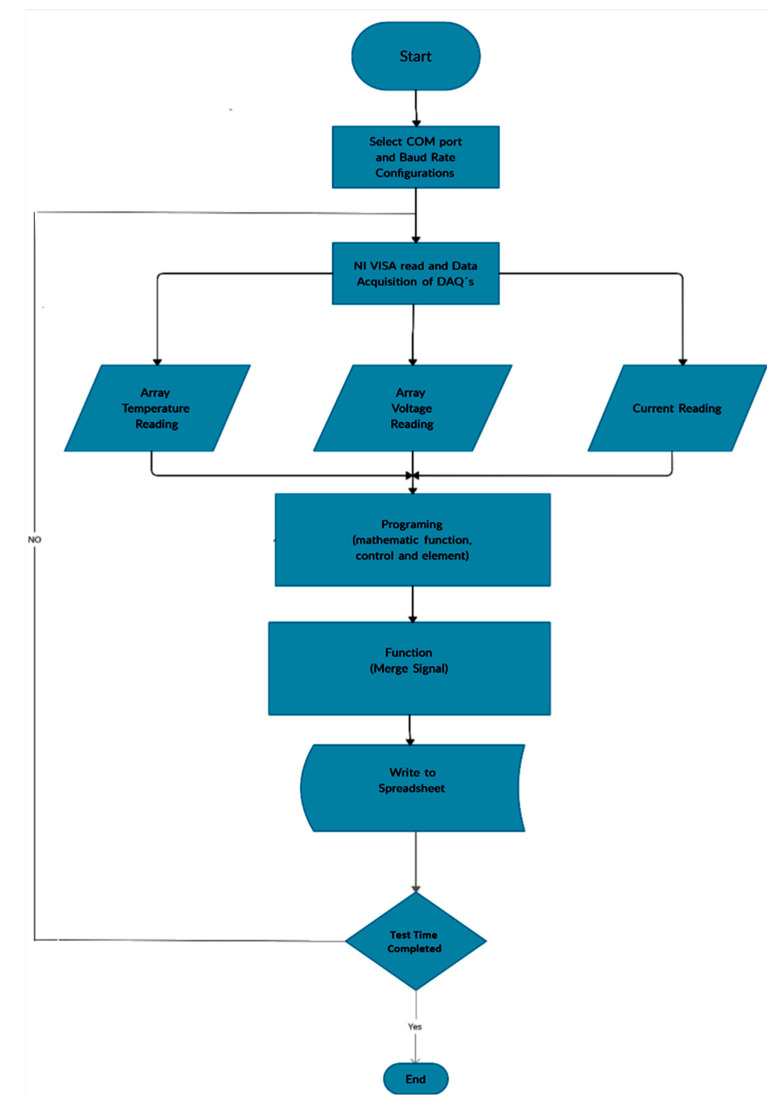
Flow chart for the designed program.

The flow chart in Figure 9 represents the sequence of operations of the acquisition system. The first block corresponds to the configuration of communication parameters. Once this is accomplished, the National Instruments VISA application allows communication between the DAQs, which allows different variables to be read from each configured DAQ to obtain temperature, voltage, current, etc. Next, a routine that includes different operations and functions that correct and adjust the different obtained signal values is executed. This loop keeps repeating until the assigned test time is completed. The values of the stack and cell voltages, the temperatures, and the current of the fuel cell are processed and plotted on a spreadsheet, and are also shown on the respective indicators and graphs of the interface.

As shown in the flow chart in Figure 9, the data are saved to a spreadsheet in.xlsx format, accessed via Microsoft Excel from the Microsoft Office Suite, and stored on the computer’s onboard storage. For each test, the user is requested to enter a specific test name, which will be used as a reference for the tests to be conducted. This storage is crucial for short- to long-term logs that can then be used to observe the behaviour of these generation systems and their degradation over time, among other possibilities.

## 5. Discussion

**Figure 10:**
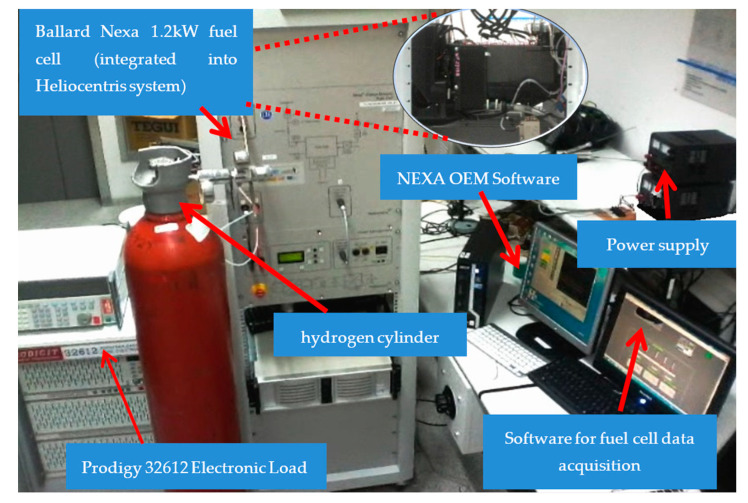
Test bench as configured for the experiments.

This section describes the different types of real-time data acquisition and visualization methods in the different tests, using the designed monitoring system to verify their functionality. The test bench used includes a hydrogen storage tank, a Ballard Nexa 1.2 kW PEM fuel cell, two computers, two benchtop power supplies, and a precision mustimeter used in [6]. The Heliocentris system was leased from the National Hydrogen Centre at Puertollano, Spain. The equipment used is shown in Figure 10.

**Figure 11:**
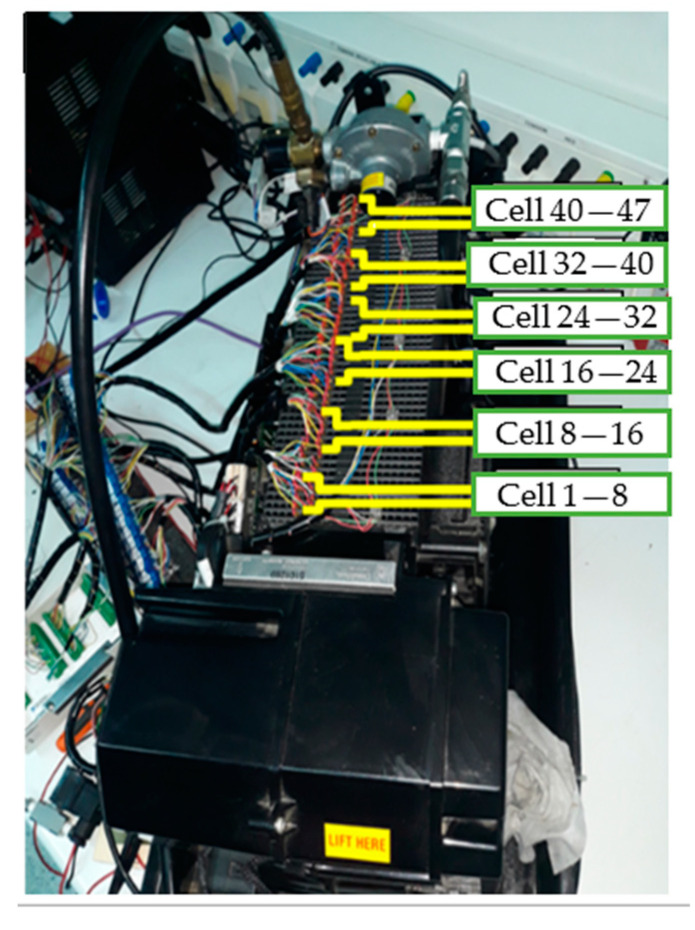
Per-cell connections across the fuel cell.

The physical connections were made non-intrusively along the stack, as shown in Figure 11.

**Figure 12:**
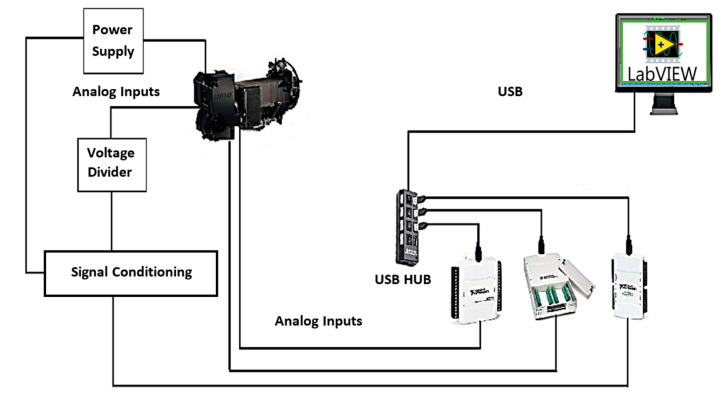
Block diagram of the test setup.

The characteristics of the fuel cell used are detailed in Table 1, and the acquisition system block diagram is shown in Figure 12.

**Figure 13:**
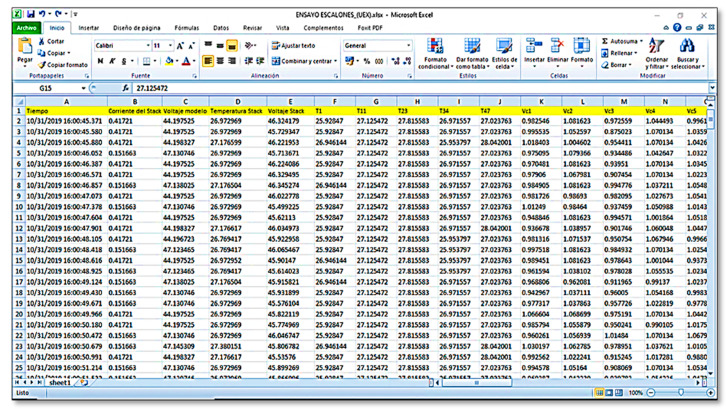
Spreadsheet log to store test generated data.

The developed system was capable of real-time measurements in external load and open loop operation modes. Figure 13 shows the table generated by the monitoring system, which was configured with different headings. The headings describe the variables to be measured, and include dates and timestamps in the following format: month, day/year: hours; minutes; seconds.

**Figure 14:**
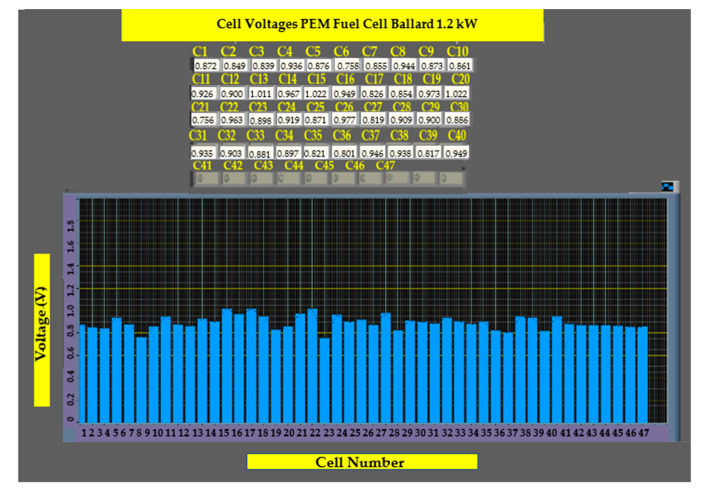
GUI showing open circuit operation.

Figure 14 shows a screenshot of the user interface, which shows the open-circuit voltage distribution across each of the cells that comprise the electrochemical system studied. The monitoring system is completely functional and has been used to perform various tests under several different conditions to validate our design.

**Figure 15:**
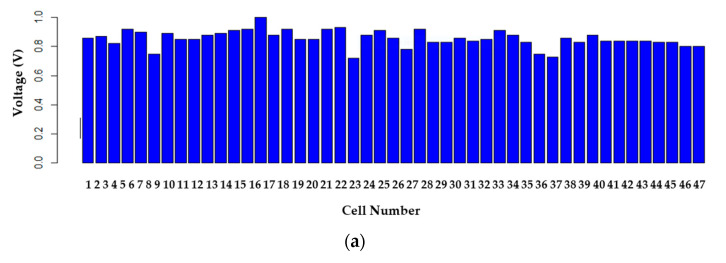

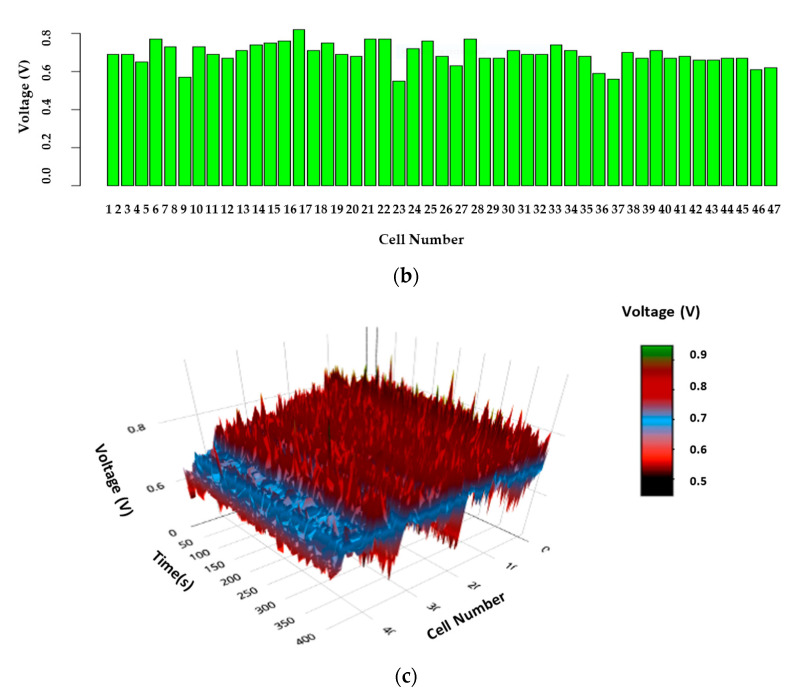
Evolution of cell voltages: (**a**) open circuit, (**b**) with external load, (**c**) 3D graph (with external load).

It shows that voltages are sustained over time and that no negative voltages exist. The evolution of voltages in Figure 15a–c shows a nonlinear distribution of voltages across the fuel cell, as the input hydrogen and oxygen are not evenly distributed or because of different chemical characteristics of cells that may improve or degrade its efficiencies, such as membrane humidity, physical degeneration, material composition, and conductivity, among other factors. This causes some cells to have less voltage and, thus, lower efficiency.

**Figure 16:**
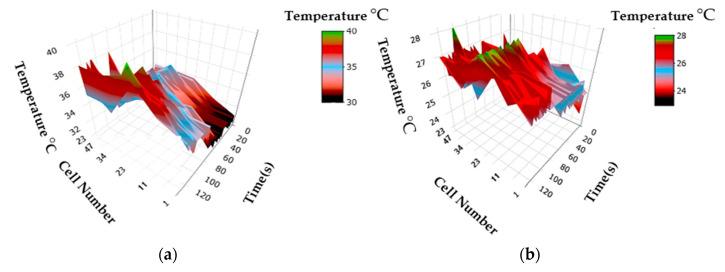
3D cell temperatures: (**a**) under 5A load, and (**b**) under no load condition.

Figure 16 shows the use of the prototype to verify the behaviour of temperatures along the hydrogen cell, applying an external load of 5 A and then no load, specifically highlighting cells 1, 11, 23, 34, and 47. It can be seen that when applying external load, there is an increase in the temperatures across the entire fuel cell. This is intertwined with the increase in chemical reactions that occur at the cell level, since generating a higher power output implies an increased consumption of fuel and the rising of ion transfers in the oxidation/reduction reactions in each of the 47 cells. On the other hand, both graphs demonstrate higher temperatures in the middle region of the fuel cells, which coincides with increases in the voltage generated in these areas, and, therefore forms the region of higher conductivity and efficiency.

## 6. Conclusions

A real-time multichannel monitoring architecture capable of measuring voltages generated in each of the 47 cells that comprise the stack of a proton exchange membrane fuel cell, as well as monitoring temperatures across the stack and output current, has been designed and constructed. Its functionality was verified by conducting tests on a Ballard Nexa 1.2 kW fuel cell that has 47 fuel cells connected internally in series.

In addition, a mass isolation PCB has been designed and developed to allow cell-by-cell voltage measurements in fuel cells with a series cell connection.

The measured cell voltages constitute a crucial parameter in the study of these systems. The developed prototype possesses a GUI with indicators that show per-cell voltages as well as fields showing maximum and minimum cell voltages separately. This information allows one to see which cells have the highest and lowest operating efficiencies in the stack, which shows how indispensable it can be as a tool for the detection and isolation of faults in the cells.

The monitoring platform was constructed with the integration of three DAQs and was programmed using NI LabView. Two signal conditioning modules were built specifically for this project, using ISO 124P isolation amplifiers, A_S-1WR2 DC–DC converters, and the miscellaneous passive components required to make them work, such as capacitors and diodes. These boards are an absolute necessity to overcome limitations normally posed by the common mode voltage of the DAQs.

The experimental results show the feasibility of the monitoring system and highlight its effectiveness in verifying the performance of proton exchange membrane fuel cells in diverse reversible (as well as irreversible) operating conditions, both in steady-state and transitory tests.

The data logging feature included serves as an invaluable tool for the detection and long-term monitoring of the health of a PEM device, which then allows for its characterization and control, among other applications.

Among the future works that can be developed from this monitoring system is the integration of a control system that allows a variety of different operating parameters to verify it. The data obtained with our monitoring system are used to build models with which the behaviour of these systems at different points of operation can be studied in greater depth, for the detection of failures, diagnoses, and forecasts of the state of health.

## Figures and Tables

**Table 1:** Main characteristics of the Ballard Nexa 1.2 kW fuel cell.

Parameter	Value
Number of cells	47
Rated voltage	26 V
Rated power	1200 W
Electrode area	115.8 cm^2^
Weight	13 kg (29 lbs.)
Length × Width × Height	56 × 25 × 33 cm

## Data Availability

Data sharing not applicable.
